# Estimation of Stroke Volume Variance from Arterial Blood Pressure: Using a 1-D Convolutional Neural Network

**DOI:** 10.3390/s21155130

**Published:** 2021-07-29

**Authors:** Hye-Mee Kwon, Woo-Young Seo, Jae-Man Kim, Woo-Hyun Shim, Sung-Hoon Kim, Gyu-Sam Hwang

**Affiliations:** 1 Asan Medical Center, Department of Anesthesiology and Pain Medicine, University of Ulsan College of Medicine, 88, Olympic-ro 43-gil, Seoul 05505, Korea; hyemee.kwon@amc.seoul.kr (H.-M.K.); kshwang@amc.seoul.kr (G.-S.H.); 2 Biomedical Engneering Research Center, Asan Medical Center, Seoul 05505, Korea; zephyrus02@gmail.com (W.-Y.S.); jaemankims@gmail.com (J.-M.K.); 3 Asan Medical Center, Department of Medical Science, Asan Medical Institute of Convergence Science and Technology, University of Ulsan College of Medicine, Seoul 05505, Korea

**Keywords:** stroke volume variance, deep learning, prediction, CNN model, mechanical ventilation, diagnostic, machine learning, neural network

## Abstract

Background: We aimed to create a novel model using a deep learning method to estimate stroke volume variation (SVV), a widely used predictor of fluid responsiveness, from arterial blood pressure waveform (ABPW). Methods: In total, 557 patients and 8,512,564 SVV datasets were collected and were divided into three groups: training, validation, and test. Data was composed of 10 s of ABPW and corresponding SVV data recorded every 2 s. We built a convolutional neural network (CNN) model to estimate SVV from the ABPW with pre-existing commercialized model (EV1000) as a reference. We applied pre-processing, multichannel, and dimension reduction to improve the CNN model with diversified inputs. Results: Our CNN model showed an acceptable performance with sample data (r = 0.91, MSE = 6.92). Diversification of inputs, such as normalization, frequency, and slope of ABPW significantly improved the model correlation (r = 0.95), lowered mean squared error (MSE = 2.13), and resulted in a high concordance rate (96.26%) with the SVV from the commercialized model. Conclusions: We developed a new CNN deep-learning model to estimate SVV. Our CNN model seems to be a viable alternative when the necessary medical device is not available, thereby allowing a wider range of application and resulting in optimal patient management.

## 1. Introduction

Given the importance of adequate fluid management to a patient’s surgical outcome, stroke volume variation (SVV), an index of fluid responsiveness, is widely used to guide fluid therapy in patients with mechanical ventilation [1]. Optimal oxygen delivery is one of the main goals in patient management and is associated with cardiac output maximization. Individualization of hemodynamic therapy and goal-directed therapy is an emerging concept to obtain cardiac output maximization based on the fluid responsiveness prediction [2]. Evaluation of fluid responsiveness has evolved from classical fluid bolus test (infusing a small volume of fluid within a short time, discarded for harming fluid non-responders with fluid overloading) to monitoring dynamic hemodynamic parameters such as SVV, which is a quantification of the respiratory variation of stroke volume from theoretical heart-lung interaction principles [3]. Goal-directed fluid therapy guided by SVV, a dynamic hemodynamic variable, is proven to be beneficial for surgical patients [4,5,6], with numerous literatures demonstrating improved patient prognosis with SVV use [5,7,8].

SVV estimated by pre-existing commercialized models (e.g., EV1000; Edwards Lifescience, Irvine, CA, USA) is derived from arterial pulse contour analysis and is widely used in current clinical practice [9,10]. This method gained popularity as it is minimally invasive compared to esophageal Doppler or pulmonary artery catheter insertion [11] and provides continuous beat-to-beat data. However, the need for additional medical sensors limits its application to all patients with arterial line. Moreover, manual calibration and manual input of patient demographic variables may unintentionally result in an unreliable estimation of SVV [12,13]. These shortcomings may result in inaccurate measures of SVV, leading to misdiagnosis, incorrect clinical treatment, and misjudgment in anesthesia care of surgical patients.

Currently, deep learning techniques are being applied to diverse medical areas [14,15]. Of deep learning models, a convolutional neural network (CNN) has shown results in solving problems such as medical imaging classification and arrhythmia detection [6,16,17,18,19,20]. A recent study reported the successful usage of CNN in clinical outcome prediction [21]. In the previous study by our lab, we successfully estimated stroke volume, which outperformed conventional arterial contour method in patients with hemodynamic instability [14]. Currently, this technique is applied to selected patients in our hospital, which provided more accurate information and eventually improved in patient management. 

Therefore, in this study, we aimed to build a novel model using the CNN technique to estimate SVV, utilizing the arterial blood pressure (ABP) waveform as the input data and SVV estimated via ABP-based commercialized SVV predicting models as a reference (EV1000).

## 2. Materials and Methods

**Data preparation**

This observational study was approved by the Asan Medical Center Institutional Review Board (No. 2018-1163) and the requirement of obtaining written informed consent from patients was waived by Institutional Review Board due to the retrospective design. A total of 10,194,698 data records from 624 patients who underwent general anesthesia and SVV monitoring in the Asan Medical Center from February 2018 to February 2019 were recorded. Of the 624 patients, 19 were excluded due to severe arrhythmia such as atrial fibrillation, and a further 48 with short surgical time or severe noise recorded were also excluded. Finally, 8,512,564 data records from 557 patients were enrolled in the deep learning model dataset. We distributed patients into the training (n = 210, data = 3,620,386), validation (n = 217, data = 3,944,244), and test (n = 130, data = 947,954) data sets. The training and validations data sets were used to build the SVV estimation model, while the test data set was used to compare the model performance with the existing model (EV1000). 

**Anesthetic management**

According to the Asan Medical Center standard anesthesia management protocol, biometric data of patients recorded during anesthesia surgery were collected as follows. Anesthesia was induced with thiopental sodium, fentanyl, and vecuronium. After intubation, anesthesia was maintained with sevoflurane or desflurane in a mixture of 50% oxygen/air. Vecuronium and fentanyl were continuously infused. Hemodynamic variables including radial and femoral arterial pressure and electrocardiogram (ECG), core temperature, pulse oximetry, and capnometer were constantly monitored. SVV was continuously monitored using EV1000. In patients with severe diseases, we used femoral vein catheterization and Swan-Ganz for monitoring inferior vena cava pressure and pulmonary arterial pressure.

**Data acquisition and Pre-processing**

Data were collected using a computer application of the medical record software called Vital Recorder [22]. Collected variables include electrocardiogram (ECG), ABP waveform, central venous pressure, pulmonary arterial pressure, and heart rate from Bx50 and SVV from EV1000. Given that breathing cycles occur every 6 to 8 s during mechanical respiration and EV1000 calculates SVV every 2 s, data sets are reconstructed with a 10 s ABP waveform to include at least one breathing cycle and corresponding SVV value recorded for 2-s intervals. The input data consisted of three channels. The first channel is normalized ABP waveform, by removing direct current components. The second channel was frequency data from 1 to 12.2 Hz, calculated with Fast-Fourier Transform. The last channel data was the slope of the ABP waveform. The output was the estimated SVV value of the corresponding time point. We used SVV from EV1000 as a reference. 

**CNN and Model Improvements**

**Figure 1:**
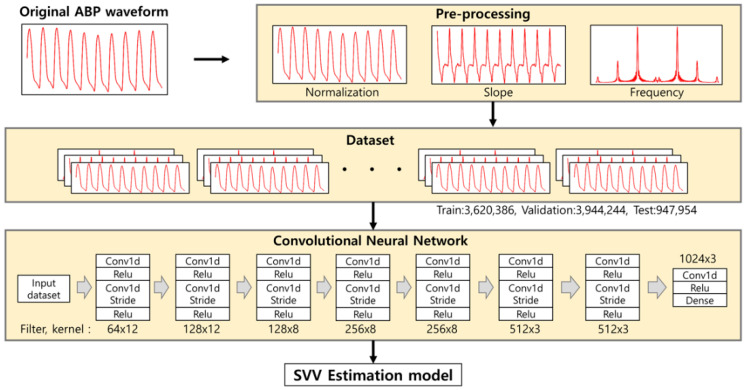
Convolutional neural network (CNN) model used in our study. The input pre-processed 10 s of arterial blood pressure (ABP) waveform into 3 channels, which consisted of pre-processed, frequency, and slope of the ABP waveform. The model consisted of 15 CNN layers and applied convolutional stride every second.

We customized our model based on the referential CNN model by VGGNET [23]. The model was built as follows. First, input sequence is 1000 samples obtained at the 100 Hz sampling rates (10 s). The dataset was collected during the entire surgical period and recorded for every two-seconds. The model consisted of 16 convolutional layers and a single fully connected layer. For direct current (DC) offset, we converted a signal from time domain to a representation in the frequency domain using Fast Fourier Transform, and removed the values in 0 hertz [24]. For dimension reduction, every two convolutional layers used one convolutional strides layer [25]. The length of the input variable starts with 1000 and halves with each convolutional stride layer. After dimension reduction, filters started at 64 and increased continuously until 1024. The kernel size started at 12 started decrease continuously to 3 (Figure 1). 

Setting up hypervariables on CNN models was done as per the temporal convolutional networks experimental guidelines [26]. Considering memory and learning time, we set the batch size to 64 and the leading rate to 10^−5^. The loss function evaluated the mean squared error (MSE) from predicted SVV and reference SVV value. The model was trained using Adam optimization, a gradient descent method [27]. We used packages for deep leaning implemented by Keras library (https://github.com/keras-team/keras (accessed on 14 October 2020)) and Python 3.6. We trained a deep learning model using a GPU server with 4 GTX-1080Ti GPUs.

**Statistical analysis**

Variables are expressed as numbers (percentages), mean ± standard deviation, or median (interquartile range), as appropriate. The intergroup analysis was performed using the student’s *t*-test, Mann-Whitney U test, logistic regression, the analysis of variance, or Kruskal-Wallis test were used for analyzing continuous variables and χ^2^ test or Fisher’s exact test for categorical variables. Linear regression analysis (Pearson correlation) was used to provide a measure of association between estimated and reference SVV. The Pearson correlation coefficient r is ranged from −1 to +1, with a higher absolute value indicating stronger association, i.e., good performance of the proposed model. The MSE and mean absolute error (MAE) was calculated to show the difference between the values. SVV obtained with our CNN models with changes of pre-processing and dimension reduction of input variable, and EV1000 was compared using Bland and Altman method, calculating bias (mean difference between measurements) and limits of agreement (equal to bias ± 2 SDs of the bias); this calculation will yield a positive number when the SVV from the proposed CNN model is higher than SVV from EV1000. A quadrant plot with concordance rate was used for the trend analysis. A 5% margin of error was used to calculate the concordance rate. We considered a *p* value < 0.05 to be statistically significant.

**Experiment: Step-wise procedure**

First, we checked the performance according to the model type using sample data. A total of six model types were tested by combining two basic preprocessing methods (min-max normalization and DC offset) and three reduction techniques (max pooling, average pooling, and using only convolution strides).

Next, we tried to increase performance by inputting additional information. The two additional inputs we entered are the ‘frequency’ and ‘slope’ of the ABP waveform. ‘Frequency’ is information in the frequency-domain, and since it contains information about heart rate, it can be an important factor in the ejection of blood from the heart. ‘Slope’ is the time derivative of ABP waveform. In each ABP waveform pattern, the slope of the epoch in which the pressure rises is an indicator of how strongly the heart ejected blood.

## 3. Results

The characteristics of the patients are displayed in Table 1. 

Patient characteristics were not statistically different among the training, validation, and test sets. A total of 8,512,564 data records (3,620,386 training data; 3,944,224 validation data; and 947,954 test data) collected for 2364 h were analyzed in the current study. Table 2 shows the hemodynamic variables including SVV measured from EV1000 in all data sets. 

Table 3 shows the performance of model according to the applied deep learning technique. 

We added pre-processing and dimensional reduction to the basic CNN model in the sample dataset, which was consisted of training (n = 33, data = 418,121) and validation sets (n = 14, data = 204,435). Removing DC offset increased model performance (correlation = 0.83, MSE = 9.3) and dimension reduction replacing the convolutional strides further increased performance (correlation: 0.83 and 0.91; MSE: 9.3 and 6.92) (Table 3).

**Figure 2:**
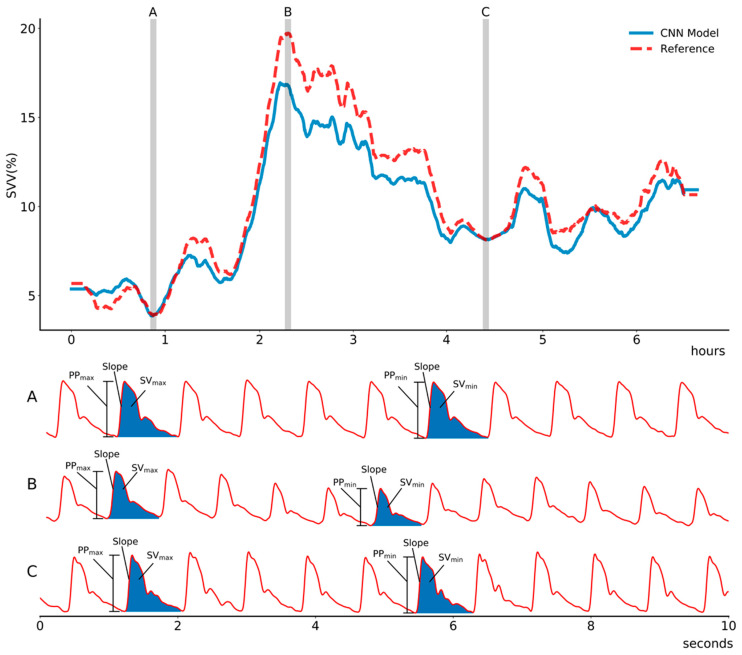
Representative plot of estimated stroke volume variance (SVV) using the proposed convolutional neural network model compared with that of a reference (EV1000, Edward, LifeScience) in (**A**) stable phase, (**B**) hypovolemic state due to massive intraoperative bleeding, and (**C**) recovery phase during intraoperative period. The proposed model shows high correlation to the reference value. Abbreviation: PP_max_, maximum pulse pressure during one ventilation cycle; PP_min_, minimum pulse pressure during one ventilation cycle; SV_max_, maximum stroke volume during one ventilation cycle; SVmin, minimum stroke volume during one ventilation cycle.

Table 4 shows model improvements (i.e., increase in Pearson correlation coefficients) according to the diversified inputs of the ABP waveform. Inputs of pre-processed and slope of ABP waveform showed a good performance, assessed by the high Pearson correlation coefficients (r = 0.93 and 0.93, respectively). The combined inputs of pre-processed and frequency signals showed improved performance, shown by increased Pearson correlation coefficients to 0.95. Figure 2 is the representative plot of the SVV derived from our model and EV1000.

**Figure 3:**
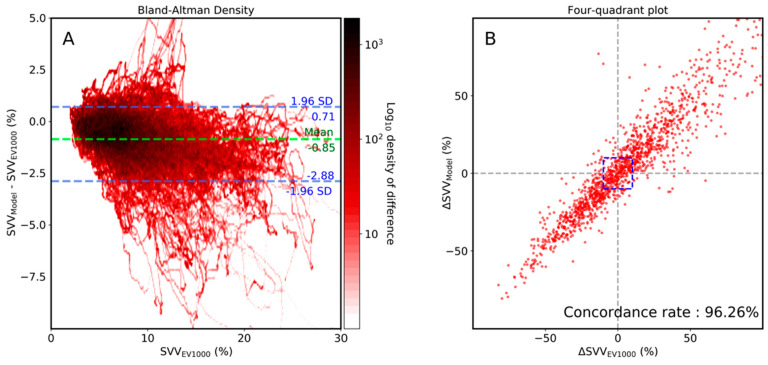
Bland-Altman density plot (**A**) and four-quadrant (**B**) plot analysis between stroke volume variance (SVV) of the proposed model and that of EV1000 (Edward, LifeScience). The central blue zone is excluded in the four-quadrant plot.

To assess the agreement between the SVV from proposed CNN model and that of EV1000, Bland-Altman analysis was performed. The mean difference (bias) was small (Bias: −0.85, 95% CI, −2.88–0.71), which indicates that the difference between SVV from the proposed CNN model and EV1000 was small. In the four-quadrant plot, the proposed CNN model’s trending ability to track SVV from EV1000 was assessed. The concordance rate from the four-quadrant plot was 96.26%, indicating the good trending ability of our proposed CNN model (Figure 3).

In the deep learning model, errors (MAE) and MSE are used to measure the model performance. Model with ABP waveforms and frequency as input showed high errors (MAE = 1.55 and 1.52, respectively; MSE = 4.74 and 5.08, respectively), whereas inputs with the pre-processed and slope of ABP waveforms showed lower errors (MAE = 1.30 and 1.38, respectively; MSE = 4.08 and 4.59, respectively). Combination of pre-processed and slope of ABP waveforms and all input vectors showed the lowest error rate (MAE: 1.24 and 1.01, respectively; MSE: 3.18 and 2.13, respectively). 

## 4. Discussion

In this study, we estimated SVV through a deep-learning approach, using arterial blood pressure waveform solely as the input. Our proposed CNN model estimated SVV with an accuracy comparable to the widely used commercialized model (EV1000). Of note, our proposed method did not require additional devices, nor manual calibration or manual input of patients’ data, which may be associated with unreliable estimation of SVV. However, our model is yet to be tested in real-time general anesthesia surgery for clinical application.

The proposed CNN model may be utilized to overcome the limitations of current commercialized models. Although the benefit of SVV is well-documented, currently clinicians must decide whether to use additional medical equipment (e.g., EV1000) to obtain SVV. Therefore, even if a patient had arterial line, SVV information might not be available if additional medical equipment was not applied or unavailable. In general surgery, clinicians decide on the extent of monitoring according to the patients’ general condition and the complexity of surgery. However, unanticipated intraoperative hemodynamic instability due to surgical manipulation or massive bleeding may occur, and the fluid management of such patients would be enhanced with a dynamic hemodynamic variable such as SVV. Our model can obtain SVV values by implanting it to the device which obtains ABP waveform, solving the spatial problem. Also, our model can be economically beneficial to patients. The medical device with a commercialized model is costly, and patients are required to pay for the one-time-use disposable medical supply products to obtain SVV measurement. One of the advantages of using our deep learning model to calculate SVV is that an additional expense or medical device is not required. Therefore, with these deep learning model-based measurements, patients who did receive SVV-based fluid management will receive better anesthesia management with SVV monitoring without any additional financial burden The merit of the proposed algorithm is that it would not require additional monitoring devices. For example, our algorithm can be applied as add-on to any monitors with arterial waveform or even could be implanted into currently wide-used bedside monitors, such as Philips, GE, and Mindray. It would provide clinicians with real-time SVV without additional burden from medical cost or device and allows SVV to be much more easily accessed and more widely used, consequently assist more rational and informed decisions regarding fluid management. Our deep learning model used a CNN technique. In the area of signal analysis, most deep learning models use recurrent neural network models, such as Long Short Term Memory [19,28]. We applied Long Short Term memory to estimate SVV to our sample dataset. However, it took a very long time and did not perform well [29]. We also tested the repeated convolutional neural network (RCNN) model, but the performance was also low. The best performance was observed for the repeated CNN strides model with dimension reduction. The strong point of the recurrent neural network technique is that it can extract pattern consideration of time continuity, while CNN is more specialized in feature extraction but has less reflection on time continuity than RNN series models [29]. It may be speculated that these differences lead the CNN technique to be more suitable in our model compared to other well-known deep-learning techniques, since it was more important to analyze the signal itself than considering the time continuity in our study.

In a typical CNN model, normalization is performed by applying the min-max method to input data [6,16,17,18,19,20,30]. However, when analyzing ABP waveform, min-max normalization may unintentionally lose valuable features that a waveform has. Therefore, the DC offset of the signal was removed without applying the min-max normalization. Moreover, max pooling, typically applied to CNN modelling, caused a problem with the ABP waveform. When processing the medical image analysis with deep-learning, high values often have essential information. However, in ABP signals, information is often present not only in high values but also in low or medium values, therefore, max pooling was not suitable for our model. Thus, we applied a convolutional strides layer to process dimension reduction [25]. This process significantly improved performance over the other models we tried. 

Our deep learning model performance was dependent on the input data processing. Input with ABP waveform or frequency of ABP waveform alone showed low performance, whereas data pre-processing significantly improved the performance of the deep learning model. Specifically, removing the DC offset and slope of the data showed significant improvement of the model. When our model was constructed with diverse inputs of data using several pre-processed methods, the results showed that the performance gradually improved with the increase in the number of input data to the multiple channel models. It may be speculated that the effectiveness of verification, rather than adding new information, is more beneficial in reducing over-fitting. 

## 5. Conclusions

We developed a novel model for estimating SVV from ABP using deep learning method. In total, 8,512,564 SVV datasets were collected from 557 patients, each dataset consisting of SVV at 2-s intervals and ABP waveform for 10 s before each SVV. We built an CNN model with existing commercialized model (EV1000) as reference, which was improved by applying pre-processing of the waveform data, multi-channel, and dimension reduction. Compared to existing commercialized monitoring device (EV1000), our initial model showed acceptable performance with Pearson correlation r = 0.91 and mean squared error MSE = 6.92. The initial model performance was improved with ABP waveform pre-processing and input diversification such as frequency and slope of ABP, which increased the Pearson correlation to r = 0.95 and reduced the min-squared error to MSE = 2.13. Furthermore, our results showed a high concordance rate of 96.26%, compared to the pre-commercialized device (EV1000). Therefore, our new model can be applied to all environments where ABP waveform is measured without additional equipment, which may enhance the efficiency of patient management and eventually improve patient outcome.

This study has several limitations. Firstly, since the model was trained with the SVV of the EV1000 as a reference, there is a possibility that SVV might not correctly reflect volume status if there was a problem with the ABP waveform. However, we manually inspected the waveform for any abnormalities, and it is reported that SVV is a trusted variable of fluid reactivity in adults [31]. Secondly, our model should be validated with SVV calculated from a golden standard method, such as esophageal echocardiography Doppler, as a reference in a future study. Lastly, external validation with different social and ethnics were not performed. We performed internal validation in an effort to overcome the limitation; however, care should be taken in the generalization of our results.

## Figures and Tables

**Table 1:** Patient characteristics.

	Training Set (N = 210)	Validation Set (N = 217)	Test Set (N = 130)	Total Set (N = 557)	*p*-Value
**Demographics**					
Age (yrs.)	58 (49–63)	56 (45–63)	56 (41–64)	57 (47–63)	0.17
Sex (male)	145 (69.0%)	154 (71.0%)	82 (63.1%)	381 (68.4%)	0.30
Weight (kg)	65 ± 12	67 ± 13	64 ± 12	66 ± 13	0.02
Height (cm)	166 (160–171)	167 (160–172)	166 (160–170)	166 (160–172)	0.13
Body mass index (kg/m^2^)	23.4 (21.1–26.2)	24.0 (21.6–26.8)	23.4 (21.6–25.4)	23.6 (21.3–26.2)	0.12
ASA classification ^a^					<0.001
1	12 (5.7)	8 (3.7)	23 (17.7)	43 (7.7)	
2	63 (30.0)	68 (31.3)	68 (52.3)	199 (35.7)	
3	111 (52.9)	115 (53.0)	38 (29.2)	264 (47.4)	
4	18 (8.6)	25 (11.5)	0 (0.0)	43 (7.7)	
5	6 (2.9)	1 (0.5)	1 (0.8)	8 (1.4)	
**Underlying disease**					
Diabetes mellitus	55 (26.2)	56 (25.8)	26 (20.0)	137 (24.6)	0.38
Hypertension	63 (30.0)	69 (31.8)	32 (24.6)	164 (29.4)	0.36
Operation time, mins	779 (399–870)	755 (423–842)	430 (320–740)	733 (376–834)	<0.001
Emergency surgery	25 (11.9)	20 (9.2)	8 (6.2)	53 (9.5)	0.21
**Type of Operation**					
Transplant ^b^	150 (71.4)	150 (69.1)	53 (40.8)	353 (63.4)	<0.001
Major open abdominal surgery	52 (24.8)	62 (28.6)	71 (54.6)	185 (33.2)	<0.001
Major laparoscopic abdominal surgery	2 (1.0)	4 (1.8)	4 (3.1)	10 (1.8)	0.36
Minor abdominal surgery	5 (2.4)	0 (0.0)	0 (0.0)	5 (0.9)	0.02
Others ^c^	1 (0.5)	1 (0.5)	2 (1.5)	4 (0.7)	0.45

ASA classification ^a^: Classification system for assessing the fitness of patients before surgery adopted by the American Society of Anesthesiologists. (1: Healthy person, 2: Mild systemic disease, 3: Severe systemic disease, 4: Severe systemic disease that is a constant threat to life, 5: A moribund person who is not expected to survive without the operation). Transplant ^b^: Liver, kidney, and pancreas transplant. Others ^c^: Postoperative bleeding control and sternal closure. Values are expressed as the mean (±SD) or median (interquartile range) for continuous variables, and n (%) for categorical variables. *p* values were calculated with analysis of variance, or Kruskal-Wallis test for continuous variables and χ^2^ test or Fisher’s exact test for categorical variables. Null hypothesis is that there is no difference of mean or median between the groups and alternative hypothesis is that there is difference of mean or median between the groups.

**Table 2:** Hemodynamic variable of training, validation, and test sets.

	Training Set (N = 210, 37.7%)	Validation Set (N = 217, 38.9%)	Test Set (N = 130, 23.4%)	Overall (N = 557)	*p*-Value
**Duration (min)**	120,679	131,474	31,598	283,752	
**Blood pressure (mmHg)**					
Systolic	110.7 ± 16.8	114.2 ± 17.4	113.0 ± 18.1	112.8 ± 17.4	<0.001
Diastolic	55.2 ± 9.6	57.5 ± 9.9	56.2 ± 9.9	56.5 ± 9.8	<0.001
**Heart rate (bpm)**	82.0 ± 15.2	81.3 ± 16.2	82.8 ± 14.1	81.8 ± 15.5	<0.001
**Stroke volume (mL/beat)**	87.3 ± 29.4	86.6 ± 26.4	80.0 ± 24.2	85.2 ± 27.5	<0.001
**Stroke volume index (mL/beat/m^2^)**	50.7 ± 16.2	50.1 ± 14.5	48.8 ± 15.2	50.2 ± 15.3	<0.001
**Systemic vascular resistance (dyne∙s/cm^5^)**	854.3 ± 354.5	880.7 ± 331.8	931.4 ± 381.0	877.6 ± 349.6	<0.001
**Cardiac output (L/min)**	7.0 ± 2.6	6.9 ± 2.3	6.5 ± 2.0	6.9 ± 2.4	<0.001
**Stroke volume variance (%)**	8.1 ± 4.9	8.1 ± 4.4	9.4 ± 5.2	8.3 ± 4.8	<0.001

Values are expressed as mean ± standard deviation or number (%). SV, SVR, SVV, SVI, CO measured by monitoring devices calculated by stroke volume using radial arterial catheter (reference data, EV1000, Edward Lifesciences). *p* values were calculated with analysis of variance, or Kruskal-Wallis test for continuous variables and χ^2^ test or Fisher’s exact test for categorical variables. Null hypothesis is that there is no difference of mean or median between the groups and alternative hypothesis is that there is difference of mean or median between the groups.

**Table 3:** Correlation between SVV derived from our CNN models with pre-processing and dimension reduction changes from the input variable and SVV from the commercialized model as a reference.

Model Type	Pearson Correlation, r	Mean Squared Error
Min-max Normalization + max pooling	0.64	33.21
Min-max Normalization + average pooling	0.66	22.86
Min-max Normalization + convolutional strides	0.65	31.15
Removed DC offset + max pooling	0.80	9.59
Removed DC offset + average pooling	0.83	9.3
Removed DC offset + convolutional strides	0.91	6.92

Compared of typical CNN model pre-processing and dimension reduction with proposed model in samples. Conv, convolutional layer.

**Table 4:** Trend analysis of various pre-processing input methods.

Data Type	Linear Regression Analysis	Bland-Altman Analysis		Mean Absolute Error	Mean Squared Error	Concordance Rate (%)
	Pearson Correlation	Bias	95% Limits of Agreement			
ABP signal	0.91	−1.00	−4.47~2.48	1.55	4.74	90.14%
Pre-processed ABP	0.93	−0.87	−4.34~2.59	1.30	4.08	92.56%
Frequency of ABP	0.88	−0.88	−5.03~3.27	1.52	5.08	88.19%
Slope of ABP	0.93	−1.00	−4.62~2.61	1.38	4.59	92.99%
Combined pre-processed and slope ABP	0.94	−0.93	−4.02~2.17	1.24	3.18	92.86%
Combined pre-processed, frequency and slope of ABP	0.95	−0.85	−2.88~0.71	1.01	2.13	96.26%

Based on pre-processed data, the results are compared with the stroke volume variation (SVV) of the model and the SVV of the EV1000. Preprocessed arterial blood pressure (ABP) waveform removed dimension reduction offset to remove baseline. Frequency data are the values that convert ABP to 1~12.2 Hz using Fast Fourier Transform (FFT). The slope data are obtained by differential ABP waveform.

## Data Availability

The data presented in this study are available on request from the corresponding author. The data are not publicly available due to privacy.
